# Genome Wide Binding Site Analysis Reveals Transcriptional Coactivation of Cytokinin-Responsive Genes by DELLA Proteins

**DOI:** 10.1371/journal.pgen.1005337

**Published:** 2015-07-02

**Authors:** Nora Marín-de la Rosa, Anne Pfeiffer, Kristine Hill, Antonella Locascio, Rishikesh P. Bhalerao, Pal Miskolczi, Anne L. Grønlund, Aakriti Wanchoo-Kohli, Stephen G. Thomas, Malcolm J. Bennett, Jan U. Lohmann, Miguel A. Blázquez, David Alabadí

**Affiliations:** 1 Instituto de Biología Molecular y Celular de Plantas (CSIC-Universidad Politécnica de Valencia), Valencia, Spain; 2 Department of Stem Cell Biology, Centre for Organismal Studies Heidelberg, Heidelberg University, Heidelberg, Germany; 3 School of Biosciences and Centre for Plant Integrative Biology, University of Nottingham, Nottingham, United Kingdom; 4 Department of Forest Genetics and Plant Physiology, Umeå Plant Science Centre, Sveriges Lantbruksuniversitet, Umeå, Sweden; 5 College of Science, King Saud University, Riyadh, Kingdom of Saudi Arabia; 6 Rothamsted Research, Harpenden, Hertfordshire, United Kingdom; National University of Singapore and Temasek Life Sciences Laboratory, SINGAPORE

## Abstract

The ability of plants to provide a plastic response to environmental cues relies on the connectivity between signaling pathways. DELLA proteins act as hubs that relay environmental information to the multiple transcriptional circuits that control growth and development through physical interaction with transcription factors from different families. We have analyzed the presence of one DELLA protein at the Arabidopsis genome by chromatin immunoprecipitation coupled to large-scale sequencing and we find that it binds at the promoters of multiple genes. Enrichment analysis shows a strong preference for cis elements recognized by specific transcription factor families. In particular, we demonstrate that DELLA proteins are recruited by type-B ARABIDOPSIS RESPONSE REGULATORS (ARR) to the promoters of cytokinin-regulated genes, where they act as transcriptional co-activators. The biological relevance of this mechanism is underpinned by the necessity of simultaneous presence of DELLAs and ARRs to restrict root meristem growth and to promote photomorphogenesis.

## Author Summary

Plants respond to environmental cues by modulating transcriptional circuits. One mechanism for such modulation involves DELLA proteins. They are promiscuous interactors of transcription factors and, in most cases, this interaction impairs the recognition of the DNA target sequences. Here we show that DELLA proteins are also recruited to multiple locations of the genome where they act as transcriptional coactivators, and we demonstrate how physical interaction with type-B ARRs is relevant for the regulation of meristem maintenance and photomorphogenesis.

## Introduction

Plant development is highly plastic in order to respond to a changing environment. Plants are able to trigger specific differentiation programs, promote growth over differentiation, or favour defense strategies over growth in response to environmental cues. Although the molecular mechanisms by which plants integrate environmental and endogenous signals are not completely understood, there is clearly a high degree of connectivity between the various elements of plant signaling pathways [1]. DELLA proteins represent one such common element by functioning as nuclear-localized transcriptional regulators, whose accumulation largely depends on the cellular levels of the hormone gibberellin (GA). Increased GA levels promote the GID1 receptor-mediated polyubiquitination of DELLAs and their subsequent degradation by the 26S proteasome [2].

Research published over the past 17 years has demonstrated multiple roles of DELLAs throughout development and in the response to biotic and abiotic stress. For example, genetic and genomic studies in Arabidopsis and rice have shown that DELLAs: (i) promote the maintenance of seed dormancy [3,4]; (ii) restrict cell elongation and division in almost all plant tissues and organs [5,6]; (iii) promote the gravitropic response in shoots and roots [7,8]; (iv) enhance the resistance to cold temperatures [9]; (v) set up the program to prevent photo-oxidative damage [10]; (vi) help establish the photomorphogenic program [11,12]; and (vii) activate the defense against necrotrophic fungi [13]. These observations reinforce the hypothesis that DELLAs are regulatory elements that impinge on–and modulate–multiple cellular pathways [14,15].

A likely explanation for the multiplicity of DELLAs’ roles is their promiscuous ability to interact with many transcription factor (TF) families [16–18]. In Arabidopsis, the DELLA proteins GAI and RGA were first found to interact physically with PHYTOCHROME INTERACTING FACTOR 3 (PIF3) and PIF4, two bHLH TFs of the PIF family and prevent their binding to target promoters [19,20]. Since then, several additional TFs have been found as partners of DELLA proteins [21–29], and the total number of interactors has been estimated to be above sixty [30]. Interestingly, this molecular mechanism can simultaneously explain two important features: the regulation of gene expression by DELLAs, and the long-standing observation of physiological crosstalk between GAs and other signaling pathways. However this exclusion of TFs from promoters is not a universal mode of action by which DELLAs can control gene expression and development since DELLA proteins are also found to be enriched at the promoters [31–33].

Cytokinins (CKs) and GAs are known to exert antagonistic regulation of multiple developmental processes [34]. For example, shoot apical meristem activity is restricted by GAs and promoted by CKs [35], while hypocotyl elongation in etiolated seedlings [12,36,37] and root growth [36,38,39] are promoted by GAs and repressed by cytokinins (CKs). At least two mechanisms have been proposed to account for this antagonistic action: a marginal repression by GAs of the expression of type-B ARABIDOPSIS RESPONSE REGULATORS (ARRs) (the DNA-binding TFs that mediate CK signaling) [40]; and independent transcriptional regulation of common targets [41]. However, the validity of these mechanisms to explain the antagonistic modulation of gene expression by GAs and CKs throughout development has not been demonstrated. Here we examine the genome-wide presence of a DELLA protein, RGA, in gene regulatory regions and define the putative *cis* elements that mediate the role of DELLAs as transcriptional coactivators. The biological relevance of this finding is supported by the identification of a novel regulatory module involving physical interaction between DELLAs and type-B ARRs that recruits DELLA proteins to generate transcriptionally active complexes at target loci.

## Results and Discussion

### RGA binds gene regulatory regions through multiple *cis* elements

**Fig 1:**
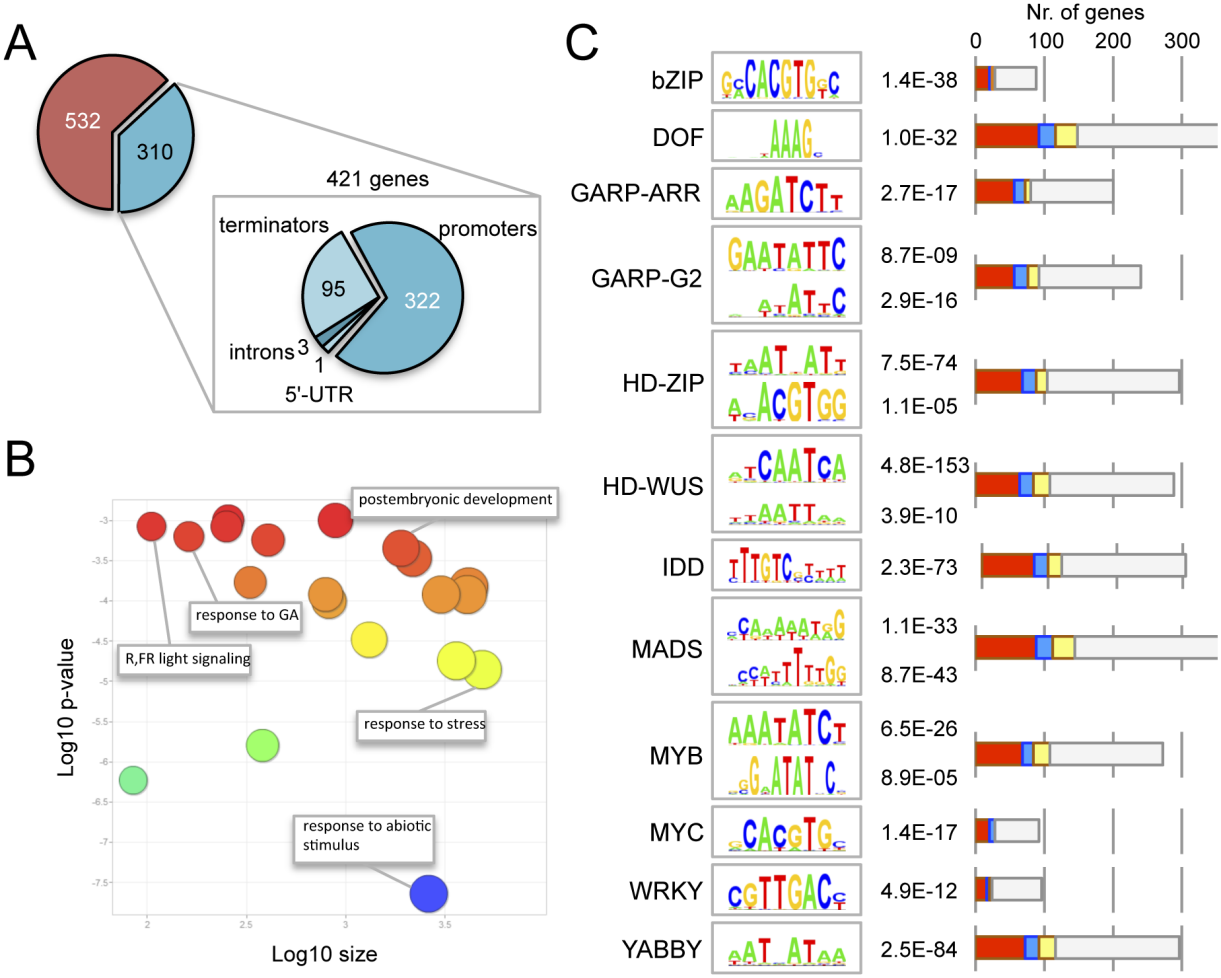
Genome-wide occupancy of RGA at target loci. (*A*) Genomic location of the statistically significant peaks of GFP-RGA along its target genes. (*B*) Gene ontology analysis of RGA targets, using ReviGO. (*C*) Statistically significant over-representation of *cis* elements for different transcription factor families. The *p* value for each element is indicated. Bars represent the number of genes with at least one copy of the corresponding *cis* element in the ChIP peak. Colours indicate induction (red), repression (blue), both (yellow) or no effect (gray) by DELLAs across all published transcriptomic datasets. Please note that each ChIP peak may contain more than one *cis* element, therefore the sum of all genes in the graph is much larger than the 421 genes associated to ChIP peaks.

To identify the *loci* at which DELLA proteins may act as transcriptional co-regulators, we incubated ten-day old Arabidopsis seedlings expressing GFP-RGA under the control of the native RGA promoter [42] in the presence of the GA biosynthesis inhibitor paclobutrazol (PAC) to induce GFP-RGA accumulation. We then performed chromatin immunoprecipitation (ChIP) with anti-GFP antibodies followed by deep sequencing (see Materials and Methods). We identified 842 reproducible binding regions. From those, only 310 could be faithfully assigned to known genes (421 genes in total) because of their proximity (2.5 kbp up- or 500 bp downstream of a gene or within introns or UTRs (Fig 1A; S1 Table). We hypothesized that this subset of genes were putative RGA targets. Gene ontology (GO) analysis indicated a statistically significant enrichment in categories related to the response to stimuli, including abiotic stress, red- and far-red light, and GA signalling (Fig 1B).

Given that DELLAs are unlikely to bind DNA directly, we were interested in identifying TFs that facilitate their association with target promoters. We examined over-represented *cis* elements within the central 200 bp of the ChIP binding regions, as most relevant *cis* elements have been reported to locate in that window [43,44]. We screened all the plant TF binding sites matrices from open-access libraries [45–47] with the MotifLab software [48], and found significant enrichment for the *cis* elements of 12 different TF families (Fig 1C), including bZIP and INDETERMINATE DOMAIN (IDD) binding sites. Interestingly, two recent reports have shown that both RGA and at least another DELLA protein (GAI) can interact with the bZIP TF ABI5 to activate the expression of the *SOMNUS* gene [49], and with several IDD family proteins to promote the expression of *SCARECROW-LIKE3* [33]. This supports the biological relevance of the over-representation of at least these elements in the set of RGA targets. RGA has also been reported to act as a transcriptional co-activator through physical association with SQUAMOSA PROMOTER BINDING-LIKE9 (SPL9) at the promoter of *APETALA1* [50], and SPL-binding sites were also enriched in the set of RGA targets in seedlings, but with very low statistical support (S2 Table). It is likely that the enrichment of DELLAs at the promoters of flowering-related genes occurs at a much later developmental stage.

The activity of DELLAs as transcriptional co-activators is also supported by two additional observations. First, the meta-analysis of published transcriptomic data involving DELLAs [14,17] indicates that the 421 genes associated with RGA ChIP peaks are preferentially induced (and not repressed) by DELLAs (see Fig 1C for a breakdown of expression behaviour depending on the enrichment of specific *cis* elements); and, second, DELLAs have been found to activate transcription in heterologous systems [51] and it has been proposed that interaction with the GID1 GA-receptors masks this activity in rice as one of the mechanisms by which GAs antagonize DELLA function [52].

### GAI and RGA interact with type-B Arabidopsis response regulators

**Fig 2:**
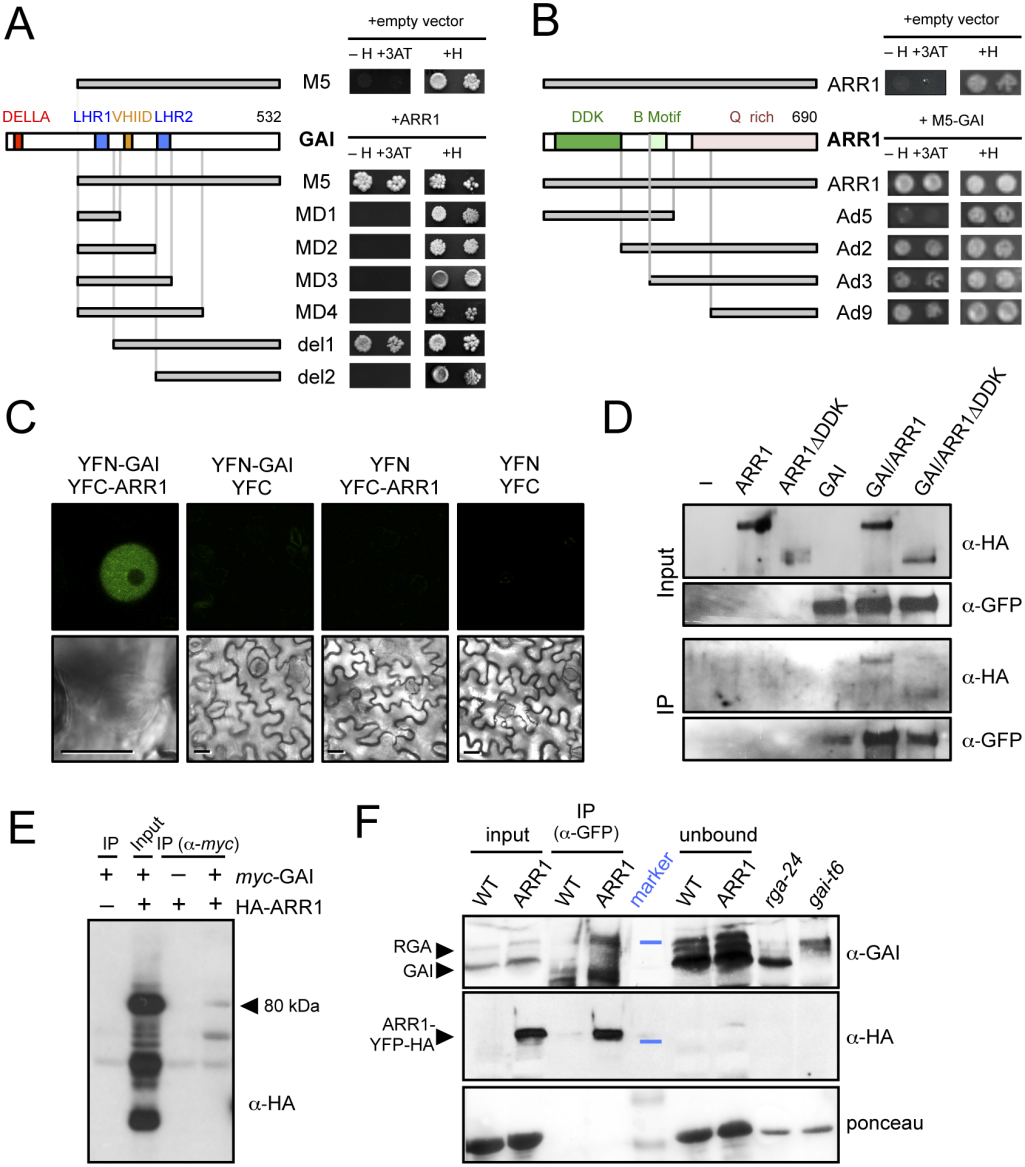
ARR1 and GAI interact physically in plants. (*A*) Y2H assay of the interaction between ARR1 and truncated versions of GAI. (H, Histidine; 3-AT, 5mM 3-aminotriazol). (*B*) Y2H assay of the interaction between M5-GAI and truncated versions of ARR1. (H, Histidine; 3-AT, 5mM 3-aminotriazol). (*C*) Bimolecular Fluorescence Complementation assay of the interaction between GAI and ARR1 in agroinfiltrated *N*. *benthamiana* leaves. Size bars, 10μm. (*D*) Analysis of the interaction between HA-ARR1 or HA-ARR1ΔDDK with YFP-GAI by co-immunoprecipitation (co-IP) with anti-GFP in agroinfiltrated leaves of *N*. *benthamiana*. (*E*) Co-IP assay of the interaction between *myc-*GAI and HA-ARR1 in *Arabidopsis* protoplasts. The arrowhead indicates the size of the expected HA-ARR1 band. (*F*) Co-IP showing the interaction between RGA and ARR1-YFP-HA in *Arabidopsis* seedlings. Proteins were immunoblotted and consecutively detected with anti-GAI and anti-HA-peroxidase conjugate antibodies. The anti-GAI polyclonal antibody recognizes both GAI and RGA. Forty micrograms of soluble proteins were loaded as input and as unbound control. Soluble proteins from the null mutants *gai-t6* and *rga-24* grown for 7 days in 0.5 μM PAC plates in continuous light were used as controls. The blue lines in the marker lane indicate the position of the 64 kDa and 98 kDa bands in the upper and middle panels, respectively. Stained bands in the marker lane in the lower panel correspond to 64 kDa and 50 kDa. WT, wild-type Col-0; ARR1, *35S*::*ARR1-YFP-HA*.

To find additional evidence for the physiological relevance of the enriched *cis* elements among RGA ChIP peaks, we scanned a comprehensive list of DELLA interactors [30] and found that twelve of them represented TF families with reported preference for binding to the enriched elements, including GARP-ARR, HD-ZIP, and MYB among others (S3 Table). The fact that not only ARR14, but also other type-B ARRs had appeared in yeast two-hybrid (Y2H) screenings performed in our labs using GAI and RGA as baits (Fig 2A and S1 Fig), prompted us to investigate (1) if DELLAs would act as transcriptional co-activators of these TFs, and (2) if these interactions could underlie the crosstalk between GA and CK signaling. Importantly, the identified ARRs (ARR1, ARR2 and ARR14) could indistinctly interact with both DELLA proteins. This result is in tune with the idea that the diversification of DELLA function relies primarily in their expression patterns, rather than in differential biochemical activities [53], also supported by the observation that all DELLA interactors analyzed to date do not show a preference for particular DELLAs. Further analysis by Y2H showed that complete removal of the LHR1 motif of GAI (del1) does not impair interaction with ARR1, whereas it was prevented by further deletion of the VHIID motif (del2; Fig 2A). These results contrast with the requirement of the LHR1 to sustain interaction of GAI or RGA with BZR1, PIF4, and JAZ1 [19,22,25]. On the other hand, the LHR1 domain was not sufficient for the interaction (Fig 2A), as occurs with BZR1 [24], in agreement with the requirement for the region close to the C-terminus to support DELLA interactions [54]. Indeed, point mutations in DELLA genes that create a premature stop codon close to the very end of the coding sequence and that produce truncated proteins represent loss-of-function alleles [55–59], most likely because of their incapacity to interact with downstream partners.

Contrary to what has been observed for other DELLA interactors, the DNA binding domain (B motif) of ARR1 was not involved in the interaction, while the glutamine-rich region responsible for the transactivation activity of ARR1 [60] was necessary and sufficient to sustain the interaction with GAI (Fig 2B). The modular nature of ARR1, demonstrated by the ability of the isolated B motif to bind DNA [60], and by the hyperactivity of the DDK-deleted version of ARR1, would be compatible with the model that DELLA binding to the C-terminus does not interfere with the regulation of ARR1 by CKs through the DDK domain or with the binding of ARR1 to the promoters.

To confirm that the interaction between GAI and ARR1 also occurs *in planta*, we performed both Bimolecular Fluorescence Complementation (BiFC) and co-immunoprecipitation (co-IP) assays. BiFC analysis showed fluorescence from the reconstituted YFP in the nuclei of epidermal cells of leaves of *Nicotiana benthamiana* co-infiltrated with YFN-GAI and YFC-ARR1, whereas the controls were below the threshold level (Fig 2C).

The co-IP experiments were performed in *N*. *benthamiana* leaves transiently expressing HA-ARR1 and YFP-GAI transgenes. HA-tagged versions of both full-length ARR1, and a deleted version lacking the DDK domain (ARR1ΔDDK), could be co-immunoprecipitated with YFP-GAI, using an anti-GFP antibody (Fig 2D). Similarly, HA-ARR1 was also co-immunoprecipitated with an anti-*myc* antibody in *Arabidopsis* protoplasts co-transfected with *myc*-GAI (Fig 2E). Remarkably, the endogenous RGA was pulled-down by anti-GFP antibodies from extracts of transgenic *Arabidopsis* seedlings expressing ARR1-YFP-HA (Fig 2F). The absence of GAI in the immunoprecipitated complexes might be a consequence of the stringent conditions and likely reflects a weaker interaction between this DELLA and ARR1 in the conditions tested. These results support the Y2H studies, demonstrating that the interaction between the DELLA proteins GAI and RGA and ARR1 occurs in plant cells, and that the DDK domain of ARR1 is dispensable for the interaction (Fig 2B and 2D).

### GAI and RGA enhance the transactivation ability of ARR1

**Fig 3:**
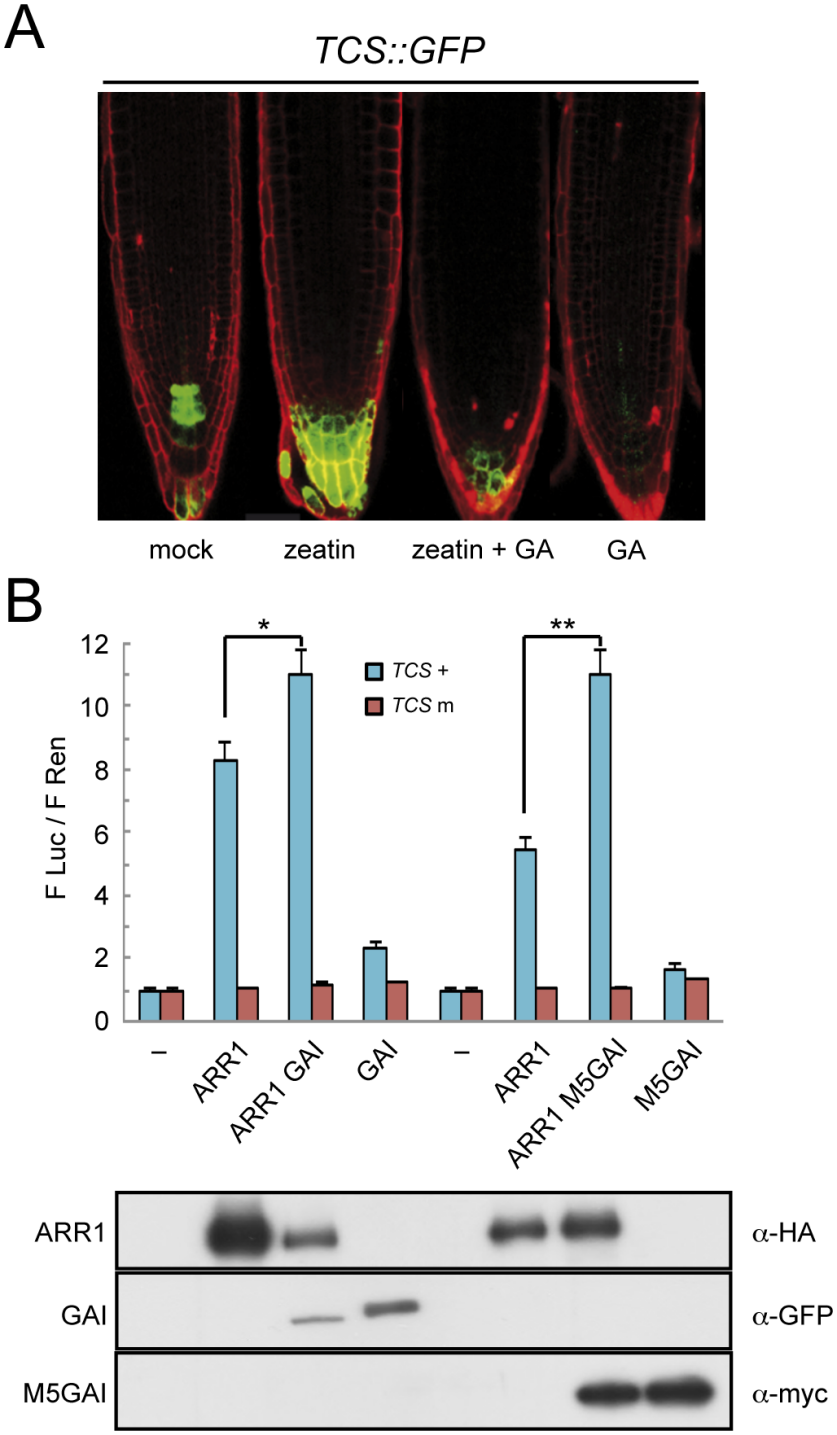
DELLAs promote ARR1 activity. (*A*) Expression in *Arabidopsis* roots of GFP under the control of the CK- and ARR1-responsive TCS element, after treatments with 0.5 μM *trans*-zeatin and 1 μM GA_4_. (*B*) Luciferase assays in *N*. *benthamiana* leaves agroinfiltrated with HA-ARR1, YFP-GAI, and *myc*-M5GAI, using the *LUC* gene under the control of the wild-type and mutant versions of the TCS element, and the constitutively expressed *Renilla* luciferase (*REN*) for normalization. The values represent the ratio between both luciferase activities and are the average of three biological replicates. Error bars are the standard deviation. One and two asterisks denote statistical significance (p<0.05 and p<0.005 respectively). The lower panel contains the western-blot analysis of the protein samples corresponding to equal mixtures from the three leaves used for the LUC assays with the wild-type TCS elements.

Given that GAs are responsible for DELLA degradation [2], and that they antagonize the effect of CKs, a reasonable hypothesis is that the interaction with DELLAs promotes the activity of the CK-activated type-B ARRs. A model in which GAs regulate the activity of ARRs is supported for example by the observation that the expression of a reporter construct in Arabidopsis roots with GFP under the control of the type-B ARR responsive TCS synthetic promoter [61] was enhanced by an 18-h treatment with 0.5 μM of *trans*-zeatin, but not in plants pretreated with 1 μM GA_4_ (Fig 3A and S2 Fig). Similarly, the same GA treatment in the absence of added *trans*-zeatin already caused a reduction in basal TCS::GFP activity (Fig 3A and S2 Fig), probably reflecting the effect on endogenous CKs. To establish whether DELLAs act as transcriptional co-activators of ARR1, we used a reporter construct containing the firefly *LUCIFERASE* (*LUC*) gene under the control of the TCS synthetic promoter and assayed its activity by transient expression in *N*. *benthamiana* leaves. As previously reported [60,61], HA-ARR1 increased the expression of the wild-type version, but not a mutated version of the *TCS*::*LUC* reporter (Fig 3B). Remarkably, the expression of *TCS*::*LUC* was significantly higher when YFP-GAI was co-expressed with either HA-ARR1 or ARR1-YFP-HA in the same leaves (Fig 3B and S3A Fig), whereas expression of YFP-GAI alone showed only a marginal increase of *LUC* expression driven by the TCS element (Fig 3B). The cooperative effect of GAI upon ARR1 activity was even more dramatic when the stabilized version of GAI (M5-GAI) was used. Importantly, higher *LUC* expression was also observed when HA-ARR1 was co-expressed with YFP-RGA, while YFP-RGA had no effect on its own (S3B Fig), indicating that the transactivation ability of ARR1 is enhanced upon interaction with at least these two DELLA proteins.

Next, we tested whether the enhanced expression of the *TCS*::*LUC* reporter upon co-expression of YFP-GAI and HA-ARR1 was due to the intrinsic transactivation of the DELLA protein. We co-expressed a truncated version of GAI, M5GAI, that still interacts with ARR1 (Fig 2A and 2B) but lacks the N-terminal part in which the transactivation activity resides [52]. As shown in Fig 3B, the activity of the reporter was enhanced when HA-ARR1 was co-expressed with *myc*-M5GAI, indicating that the enhanced transactivation is not due to the N-terminal part of the DELLA protein. These results also suggest that either the DELLA protein recruits additional transcriptional co-activators to the complex or other regions of the DELLA acquire transactivation ability upon interaction with ARR1.

### ARR1 mediates the presence of RGA at target promoters

To identify the most relevant targets for co-regulation by ARR1 and DELLAs, we chose to perform a microarray analysis on seedlings that expressed the conditional ARR1ΔDDK:GR allele under the 35S promoter [62] in the presence or absence of PAC (Silverstone et al. 2001). In these seedlings, a treatment with dexamethasone (DEX) causes translocation of ARR1ΔDDK to the nucleus, where it regulates the transcription of its target genes. Therefore, we searched for genes displaying differential expression after a 3-h treatment with 5 μM DEX depending on the presence of 10 μM PAC (see Materials and Methods for details). In parallel, we also examined transcriptomic changes induced by 5 μM N6-benzyladenine (BA) both in the presence and in the absence of PAC, to identify those targets in which regulation by CKs would be primarily dependent on ARR1.

**Fig 4:**
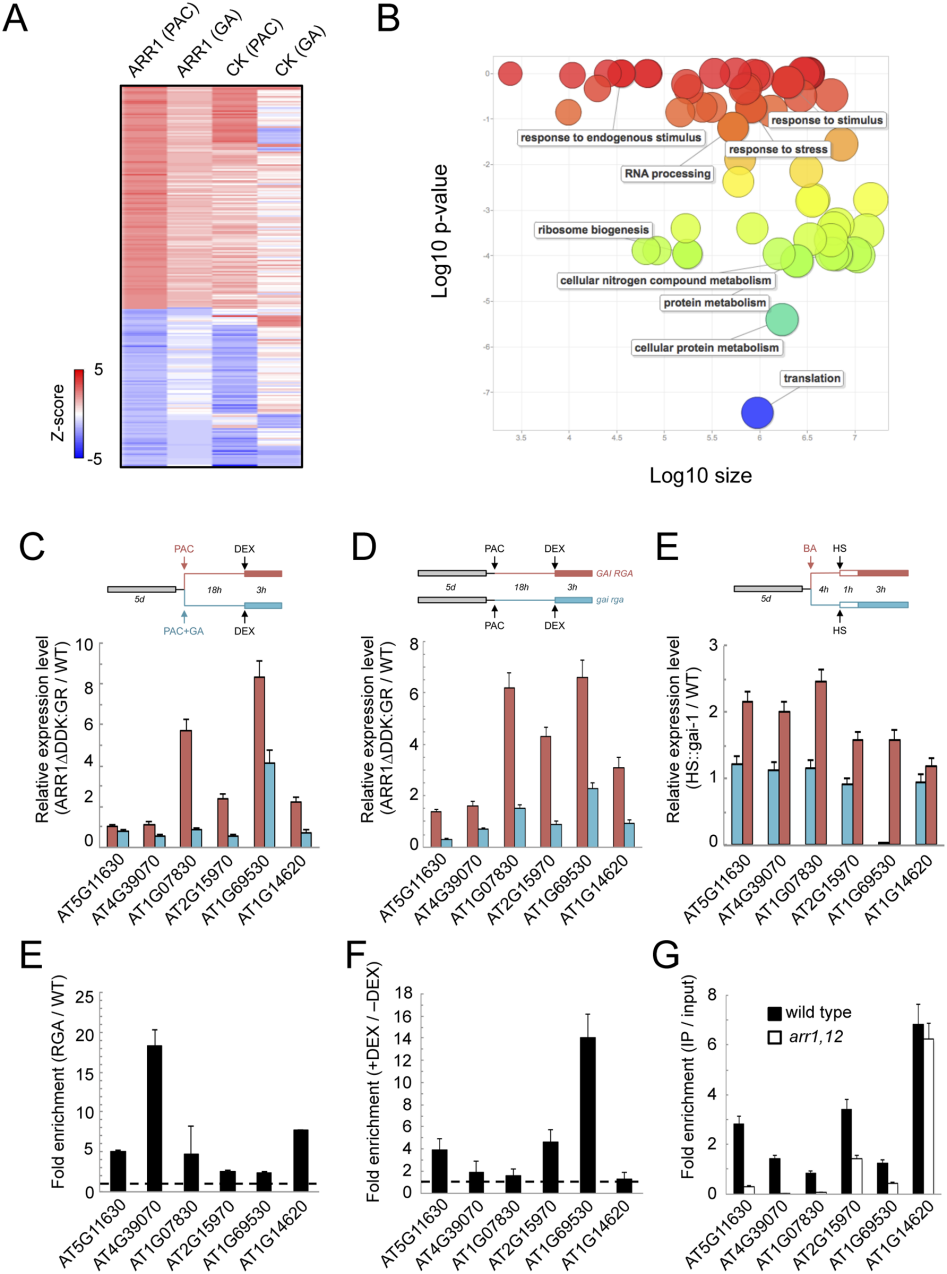
ARR1 and DELLA act as transcriptional co-regulators in Arabidopsis. (*A*) Heat map representation of the *Arabidopsis* gene set whose regulation by ARR1ΔDDK:GR or CKs depends on DELLA proteins. The colour scale represents Z-scores. (*B*) Enrichment of GO categories of all ARR1 target genes in the presence of DELLAs, visualized with ReviGO. (*C*) Gene expression analysis by RT-qPCR in response to short-term ARR1ΔDDK:GR induction with or without PAC. (*D*) Gene expression analysis by RT-qPCR in response to short-term induction of gai-1 with or without BA. (*E*) ChIP analysis of RGA::GFP-RGA at the promoters of six representative common targets for ARR1 and DELLAs. (*F*) Fold enrichment of GFP-RGA at selected target promoters in the presence (+DEX) vs the absence (-DEX) of ARR1ΔDDK:GR, in F1 seedlings of a cross between *RGA*::*GFP-RGA* and *35S*::*ARR1ΔDDK*:*GR* plants. In this experiment, qPCR values of ChIP samples were normalized per input in each condition (-DEX, and +DEX), and here we show the ratio between those two conditions. (*G*) ChIP analysis of endogenous RGA at the promoters of six representative common targets for ARR1 and DELLAs, in the wild type and in *arr1 arr12* mutants. ChIP was performed with anti-RGA antibodies. For (*C-G*), data correspond to single biological samples analyzed in triplicates. A second biological sample showed equivalent results.

Statistical analysis of the transcriptome data by *Z-score* transformation [63,64] revealed 638 genes were up-regulated and 1070 down-regulated by activated ARR1. From those, only 99 were still up-regulated both under high or low DELLA levels, and included well-known targets of CK signaling, like type-A *ARR* genes, and CK Response Factors (S4 Table). Most interestingly, 140 genes were identified whose expression was induced by ARR1ΔDDK only when DELLA levels were high, while 99 genes were repressed in those conditions (Fig 4A). GO analysis of the genes induced by ARR1 preferentially in the presence of DELLAs indicates a statistically significant enrichment of several categories, with a preference for ribosome biogenesis, translation, and protein metabolism (Fig 4B).

Among the genes differentially expressed in the presence of both DELLAs and ARR1, only four displayed a statistically significant ChIP peak for RGA (S1 Table). This low overlap probably reflects the difference in the experimental set-up. Given that ARR1 can act as a transcriptional activator, we selected six of the induced genes to further test the functional and molecular relationship between ARR1 and DELLAs. First we examined the consequence of short-term activation of ARR1ΔDDK:GR in light-grown seedlings that had high or low levels of DELLA proteins. Four of the six genes showed a much stronger induction by ARR1ΔDDK in seedlings with high DELLA levels (Fig 4C), in agreement with the global transcriptomic analyses performed under similar conditions. Then we did the reciprocal test in which we examined the influence of an activated CK pathway on the ability of gai-1 to induce gene expression using *HS*::*gai-1* seedlings [65]. In this case, five of the six genes displayed a stronger induction in gai-1 seedlings that had been pretreated with 5 μM BA, than in the untreated plants (Fig 4D), which supports the idea that type-B ARRs and DELLAs jointly promote transcription of the target genes.

If the co-regulation of the target genes by DELLAs and ARR1 is mediated by physical interactions between these two proteins, then DELLAs should be present at the promoters of these particular targets in an ARR1-dependent manner. To test this prediction, we first performed ChIP on *RGA*::*GFP-RGA* seedlings. In fact, the presence of RGA was significantly enriched in the promoters of the six genes tested (Fig 4E) and, what is more important, the presence of GFP-RGA at the promoters of three of the six genes tested was much higher in seedlings when ARR1ΔDDK:GR accumulated in nuclei after DEX treatment (Fig 4F). The requirement for ARR1 in the binding of RGA was further supported by the loss of enrichment in the *arr1 arr12* double mutant, compared with the wild type, in some of the *loci* examined (Fig 4G). Our results suggest that ARR1 mediates the binding of DELLAs to the target promoters, and together they promote the expression of target genes.

### DELLA-ARR1 interaction is necessary for proper root meristem maintenance and skotomorphogenesis

**Fig 5:**
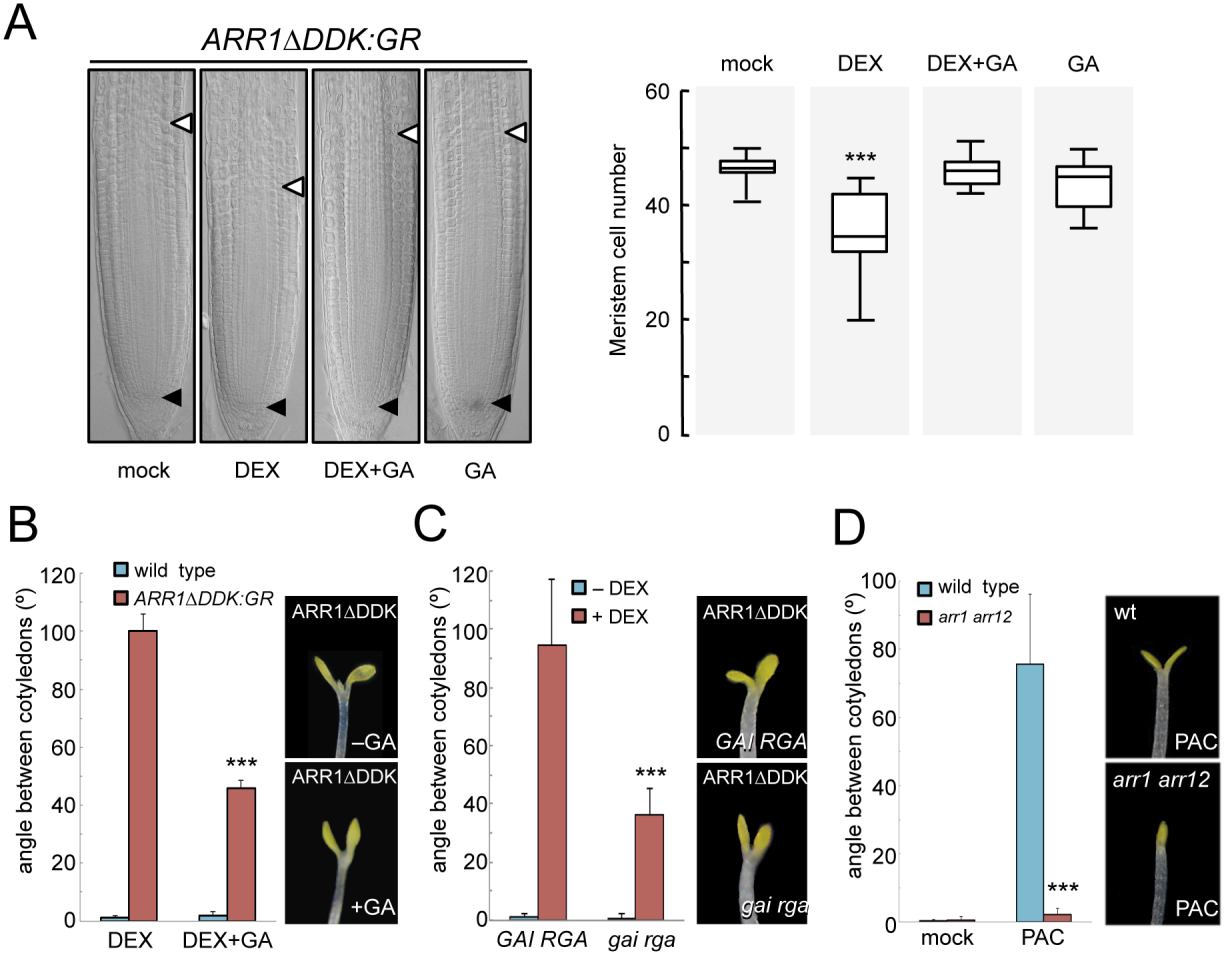
Physical interaction between ARR1 and DELLAs regulates division at the root meristem and photomorphogenesis. (*A*) Root meristem size of *35S*::*ARR1ΔDDK*:*GR* seedlings grown for 4 days with and without GAs. (n = 20; data are mean ± SD, ***p<0.001 in a Student’s t-test with respect to control plants). Arrowheads mark the extension of the meristem. (*B*) Angle between cotyledons of *35S*::*ARR1ΔDDK*:*GR* seedlings grown for 4 days with and without GAs in darkness. (n = 18; data are mean ± SD; ***p<0.001 in a Student’s t-test with respect to seedlings treated with DEX without GAs). (*C*) Angle between cotyledons of *35S*::*ARR1ΔDDK*:*GR* seedlings in *gai rga* double mutant or in an otherwise wild-type background. Seedlings were grown for 4 days in darkness. (n = 15; data are mean ± SD; ***p<0.001 in a Student’s t-test with respect to *35S*::*ARR1ΔDDK*:*GR GAI RGA* seedlings treated with DEX). (*D*) Angle between cotyledons of wild-type and *arr1 arr12* seedlings grown for 4 days with and without PAC in darkness. (n = 18; data are mean ± SD; ***p<0.001 in a Student’s t-test with respect to PAC-treated wild-type seedlings). Experiments were performed as indicated in Materials and Methods. Equivalent treatments of wild-type seedlings with DEX did not cause any change in root meristem size and or the angle between cotyledons.

A physical interaction between ARR1 and DELLAs provides a likely mechanism for the antagonistic effect of CKs and GAs in the regulation of gene expression. To probe the physiological relevance of this particular mechanism in the control of plant development we decided to test the impact of altering this interaction on two processes known to be regulated both by CKs and GAs. DELLA accumulation has been shown to reduce cell division at the root meristem [38,39] resembling the arrest caused by ARR1 overproduction [66,67]. Indeed, it has been shown that ARR1 mediates the reduction of cell division via DELLAs, and the proposed mechanism involves the promotion of *ARR1* gene expression by DELLAs [40]. To test the relevance of the interaction between the ARR1 and DELLA proteins in this context and separate the possible effect on *ARR1* expression, we examined the ability of the constitutively expressed version of ARR1 (*35S*::*ARR1ΔDDK*:*GR*) to block root meristem growth depending on the presence of DELLAs. Induction of ARR1ΔDDK:GR translocation into the nucleus by DEX treatment caused a reduction in root meristem size (Fig 5A). Importantly, this effect could be completely reversed by GA treatment that depletes DELLAs from root cells (Fig 5A), indicating that this class of proteins are required for full ARR1 function, rather than for *ARR1* expression.

At a different developmental stage, exogenous CKs have been shown to promote photomorphogenesis [37], while GAs repress photomorphogenic development in dark-grown seedlings [11,12,65]. Accordingly, nuclear accumulation of ARR1ΔDDK:GR in dark-grown seedlings resulted in cotyledon expansion, a well known photomorphogenic trait (Fig 5B). This effect was milder in GA-treated seedlings, indicating that DELLAs enhance the photomorphogenic activity of ARR1. This conclusion was supported by the observation that the photomorphogenic effect caused by ARR1ΔDDK:GR induction was also attenuated in a *gai rga* null mutant background (Fig 5C), also in agreement with these two being the most relevant DELLA proteins in the control of several aspects of photomorphogenesis [12]. Conversely, the stimulation of cotyledon opening by DELLAs, achieved by PAC treatment as previously reported [11,12,65], was completely suppressed in the *arr1 arr12* double mutant (Fig 5D), supporting the idea that ARRs and DELLAs jointly regulate various physiologically relevant developmental processes.

Taken together, our results and other recent reports [33,50] expand the mechanism by which DELLA proteins regulate transcriptional programs in plants. The observation that DELLAs modulate not only the binding, but also the activity of TFs at target loci, together with the indications that they may also regulate chromatin remodeling through their interaction with SWI/SNF complexes [68] delineates a landscape in which DELLA proteins act as molecular hubs in signaling networks, with a profound effect on plant physiology. Equivalent central roles have been found in other systems only for mitogen-activated protein kinases (MAPKs). For instance, mammalian p38 kinases and their yeast ortholog Hog1 modulate gene expression in a very wide sense by regulating the activity of DNA-binding TFs, transcriptional elongation, chromatin remodeling, and mRNA stability in response to environmental stress [69]. However, the activity of DELLA proteins relies on their intrinsic ability to interact with elements of the transcriptional regulation machinery.

Under this perspective, at least two relevant issues would need to be solved: the molecular features of DELLA proteins that allow them to display such a promiscuous set of interactors and activities; and the spatial requirements that may constrain the different DELLA interactions to specific cell-types.

## Materials and Methods

### Plant material and growth conditions

*Arabidopsis thaliana* accessions Col-0 and L*er* were used as wild type as indicated. The transgenic lines *35S*::*ARR1ΔDDK-GR*, *TCS*::*GFP*, *RGA*::*GFP-RGA*, *HS*::*gai-1* and the mutants *gai-td1*, *rga-100* and *arr1-3 arr12-1* in the Col-0 background have been described previously [36,42,61,62,65,70]. To over-express *ARR1*, the open reading frame without stop codon was amplified from an *Arabidopsis* seedlings cDNA pool and cloned into the pEarleyGate-101 binary vector to create the ARR1-YFP-HA fusion. Wild type Col-0 *Arabidopsis* plants were transformed by the floral dip method. Primers used for amplification of the *ARR1* ORF are in S5 Table.

Seedlings were grown on MS at 22°C in continuous fluorescent white light (~50 μmol m^−2^ s^−1^) unless otherwise indicated. For TCS::GFP activity, 6-day-old seedlings growing in MS plates were transferred to liquid MS containing 1 μM GA_4_ (Duchefa) for 3 h and then *trans*-zeatin (Sigma) was added to a final concentration of 0.5 μM for 18 h. For root meristem growth assays, 5-day-old seedlings were incubated in liquid MS for 16 h in the presence of 30 μM dexamethasone (Sigma) and/or 1 μM GA_4_, and then transferred to MS plates for 2 days. For cotyledon opening assays, stratified seeds were incubated for 8 h in the light, and then transferred to darkness in MS plates supplemented with 0.1 μM dexamethasone (DEX) and/or 1 μM GA_4_, or with 0.5 μM PAC (Duchefa) for 7 days.

### ChIP-seq

Transgenic *RGA*::*GFP-RGA* and the control non-transgenic seeds were sown on MS plates and stratified for 4 days at 5°C. Seedlings were grown at 150 μmol m^-2^ s^-1^ for 10 days under long day conditions before being transferred to a hormone liquid treatment with 10 μM PAC for 18 h. This was the minimum incubation time required to cause an effective block in GA biosynthesis and deplete previously synthesized GAs, as indicated by the analysis of several marker genes [30].

ChIP assays were performed from 2 g of fresh weight each as previously described (Gendrel et al., 2005). Nuclear extracts were split in two and incubated each with 20 μl of GFP-Trap_A (ChromoTek) for 4 h at 5°C for immunoprecipitation. MinElute Reaction Cleanup Kit columns (Qiagen) were used for purification of the DNA fragments. Enrichment of specific DNA fragments was validated by qPCR at the *SCL3* promoter region by comparing immunoprecipitated DNA to the corresponding input sample. Three independent sequencing libraries were generated for the GFP-RGA and WT ChIP using pooled DNA from 7 to 10 individual ChIP preparations. Six independently bar-coded libraries were pooled in a single lane and sequenced by 51-cycle single-end sequencing on the Illumina HiSeq 2000 platform. Sequencing and library construction was performed by the Deep Sequencing Core Facility of the CellNetworks cluster of the University of Heidelberg.

All reads were mapped to chromosomes 1–5 of the TAIR10 genome using bowtie2 (v2.0.5) [71] with default settings of the—fast option. Identification of binding sites was performed independently for each biological replicate using MACS (v1.4.2) [72] with the following options:—nomodel—shiftsize 75—keep-dup auto-g 1.2e8-w-S. Binding summits were considered reproducible between biological replicates when located within 200 bp of each other. For each reproducible binding region a new mean summit position was calculated at the average position of the individual summits using bedtools multiinter (v2.17.0) [73] and subsequently extended equally on both sides to define a 200 bp binding site.

Annotation of bound genes was performed with the help of a toolset ("Operate on Genomic Intervals") provided by the Galaxy server [74–76] as well as the gene annotations of the TAIR10 genome. GFP-RGA-bound genes were defined as those having one or more binding sites within 2.5 kb upstream of their transcription start sites (TSS), or 500 bp downstream of the transcriptional end (TSE) with no intervening gene between the binding site and the TSS/TSE. Additionally genes were also annotated as bound by GFP-RGA when binding sites occurred within the untranslated region (UTR) or intron of a gene. By these criteria, GFP-RGA-associated genes were, in some cases, identified as having more than one binding site, and individual binding sites were found that are associated with up to two genes on opposing DNA strands.

### Yeast two hybrid assay

A cDNA library from three-day-old etiolated seedlings, prepared in the pACT vector [77], was screened by Y2H using M5GAI and RG52 (the equivalent M5 truncated version of RGA) fused to the Gal4-DNA binding domain (DBD) in the pGBKT7 vector (Invitrogen) as bait. To test truncated versions of ARR1, all constructs were made by recombining entry clones to GATEWAY destination vectors via LR Clonase II (Invitrogen). Primers used for plasmid construction are listed in S5 Table. PCR products were cloned into pCR8/GW/TOPO (Invitrogen), then transferred into pDEST22 (Invitrogen) to create Gal4-AD fusion (ARR1-GAL4DBD versions display strong activation of the HIS3 reporter on their own). GAI deletions have been previously described [24]. Yeast AH109 cells were cotransformed with specific bait and prey constructs. All yeast transformants were grown on SD/-Trp/-Leu/-His/-Ade medium for selection or interaction tests, in the presence of different concentrations of 3-aminotriazol (3-AT) (Sigma).

### BiFC analysis

pENTR clones containing the full length of ARR1 and GAI were transferred into pMDC43-YFC and pMDC43-YFN vectors respectively [78]. BiFC experiments were performed as previously described [79].

### Coimmunoprecipitation assays

Co-IP of GAI and ARR1 in *N*. *benthamiana* leaves was performed as previously described [23], using the corresponding constructs in pEarleyGate-201 (ARR1, ARR1ΔDDK) and pEarleyGate-104 (GAI) [80].

*Arabidopsis* cell suspension derived from wild type Col-0 roots was used for protoplast isolation [81]. Transfections of protoplasts were performed as described [82], with 3 μg each of myc-GAI and HA-ARR1 expression constructs. Transfected protoplast were cultured for 16 h at RT and then lysed in extraction buffer [25 mM Tris-HCl (pH 7.8), 5 mM EGTA, 10 mM MgCl_2_, 75 mM NaCl, 10% (v/v) glycerol, 0.2% (v/v) Tween-20, 2 mM DTT and 1% (v/v) plant protease inhibitor cocktail (Sigma)]. In co-IP assays, proteins were incubated in a total volume of 100 μl of extraction buffer containing 150 mM NaCl, 0.2 mg ml^-1^ BSA and 1.5 μg of anti-c-myc antibody (clone 9E10, Covance). Immunocomplexes were captured on Protein G-Sepharose beads (GE Healthcare), washed three times in 500 μl of washing buffer [1xTBS, 5% (v/v) glycerol, 0.1% (v/v) Igepal CA-630] and eluted by boiling in 25 μl of 1.5x Laemmli sample buffer. Proteins were then resolved by SDS-PAGE and blotted to a PVDF membrane (Millipore). The presence of HA-ARR1 protein was detected by a monoclonal anti-HA-peroxidase conjugate antibody (clone 3F10, Roche) with ECL Reagent (GE Healthcare).

For co-IP assays in *Arabidopsis*, *35S*::*ARR1-YFP-HA* and Col-0 wild-type seedlings were grown in MS plates at 22°C under continuous fluorescent white light (~50 μmol s^-1^ m^-2^) for 7 days, being the media supplemented with 10 μM PAC for the last 2 days. Finally, seedlings were soaked in a solution containing 10 μM N6-benzyladenine (Sigma) for 2 h. Frozen seedlings were ground with a mortar and a pestle and the resulting powder homogenized in one volume (700 μl) of cold extraction buffer [50 mM Tris-HCl pH 7.5, 100 mM NaCl, 1% (v/v) Nonidet P-40, 1 mM PMSF, and 1x complete protease inhibitor cocktail (Roche)]. Extracts were centrifuged twice for 15 min at full speed in a top bench microcentrifuge at 4°C. Total soluble proteins in the supernatant were quantified by Bradford assay. Forty micrograms of soluble proteins were saved to be used as input, and 500 μg were used for the co-IP. First the extract was pre-cleared by incubating with 15 μl of Dynabeads Protein A (Life Technologies) at 4°C for 1 h and 15 min in a total volume of 650 μl. The anti-GFP antibody (A6465, Life Technologies) was cross-linked to Dynabeads Protein A following manufacturer’s instructions (Life Technologies). Pre-cleared extracts were incubated with the cross-linked antibody at 4°C for 1 h and 40 min. Forty micrograms of unbound proteins were saved as control. Beads were washed three times with 300 μl of cold washing buffer [50 mM Tris-HCl pH 7.5, 100 mM NaCl, and 1% (v/v) Nonidet P-40]. Proteins were eluted in 70 μl of 1x Laemmli sample buffer by incubating at 95°C for 5 min. Immunoprecipitated proteins were run in an 8% SDS-PAGE, immunoblotted, and detected with anti-GAI antibodies [3]. Subsequently, blots were stripped-out and incubated with anti-HA- peroxidase conjugate antibody (clone 3F10, Roche).

### Transient transactivation assay

The reporter construct contained six copies of a sequence containing duplicate *cis* elements bound by type-B ARRs binding site in its wild-type (TCS) (AAAATCTACAAAATCTTTTTGGATTTTGTGGATTTTCTAGC) and mutant forms (TCSm) (AAAATGTACAAAATGTTTTTGCATTTTGTGCATTTTCTAGC) as reported (Müller and Sheen, 2008), upstream of the minimal *35S* promoter and the Ω translational enhancer in the pGreenII 0800-LUC vector [83]. DNA fragments containing the *cis* elements were amplified from the corresponding constructs in pUC18 [61] using the primers indicated in S5 Table. The effector constructs were prepared in pEarleyGate-201 and pEarleyGate-101 (ARR1), pEarleyGate-203 (M5GAI) and pEarleyGate-104 (GAI and RGA).

Transient expression in leaves of *N*. *benthamiana* was achieved by infiltrating mixtures of Agrobacterium cultures. The reporter:effector ratio was 1:4 for ARR1, while was 1:4 for GAI and M5GAI. Firefly and the control Renilla LUC activities were assayed from leaf extracts with the Dual-Glo Luciferase Assay System (Promega) and quantified with a GloMax 96 Microplate Luminometer (Promega). Control Western blots were performed with proteins extracted from the same experiment, and the ARR1, GAI, M5GAI, and RGA fusions were detected with anti-HA (3F10; Roche), anti-GFP (ab290; Abcam), anti-GAI [3] and anti-c-*myc* (9E10; Roche) antibodies.

### Gene expression

For gene expression analysis, total RNA was extracted with E.Z.N.A. Plant RNA Mini Kit (Omega Bio-tek) according to the manufacturer’s instructions. cDNA synthesis was performed with SuperScript II First-Strand Synthesis System (Invitrogen). qPCR was performed as previously described [84], using the *EF1-α* gene for normalization.

For microarray analyses, RNA was extracted with RNeasy Plant Mini kit (Qiagen) according to manufacturer’s instructions. RNA labeling and hibridization to Affymetrix *ATH1* arrays were performed by GeneCore facility at EMBL Heidelberg. Statistical analysis of microarray data was performed using Z-score transformation [63], and selecting differential genes with p<0.05.

### Chromatin immunoprecipitation

Ten-day-old L*er* wild type seedlings and the *RGA*::*GFP-RGA* line grown in continuous light (~50 μmol s^-1^ m^-2^) were treated with 10 μM PAC (Duchefa) for 18 h. Then, N6-benzyladenine (Sigma) was added to a final concentration of 5 μM for 6 h; a mock treatment was used as control. ChIP was performed as previously described [85], using Dynabeads Protein A (Life Technologies) and an anti-GFP polyclonal antibody (ab290; Abcam). Relative enrichment was calculated by normalizing the amount of target DNA, first to the internal control gene *HSF* (At4g17740) and then to the corresponding amount in the input. The same was done with *35S*::*ARR1ΔDDK*:*GR* x *RGA*::*GFP-RGA* F1 crosses. Data are mean and SD of three technical replicates from a representative experiment, out of the two biological replicates performed.

To examine the localization of RGA at chromatin in the *arr1 arr12* mutant background, ChIP was performed using anti-RGA antibodies [86].

## Supporting Information

S1 Fig GAI and RGA interact indistinctly with ARR1, ARR2 and ARR14. (*A*) Y2H assay of the interaction between ARR1 and a truncated version of RGA without the DELLA domain. (H, Histidine; 3-AT, 5mM 3-aminotriazol) (*B*) Y2H assay of the interaction between ARR2 and truncated versions of RGA. (H, Histidine; 3-AT, 5mM 3-aminotriazol) (*C*) Y2H assay of the interaction between ARR14 and a truncated version of GAI without the DELLA domain. (H, Histidine; 3-AT, 5mM 3-aminotriazol)
(TIF)

S2 Fig DELLAs promote ARR1 activity. Expression in Arabidopsis roots of GFP under the control of the CK- and ARR1-responsive TCS element, after treatments with 0.5 μM *trans*-zeatin and 1 μM GA_4_. Several individuals are shown, to complement the information of Fig 3A.
(TIF)

S3 Fig RGA enhances the transactivation ability of ARR1. Luciferase assays in *N*. *benthamiana* leaves agroinfiltrated with ARR1-YFP-HA and YFP-GAI (*A*), and with HA-ARR1, YFP-GAI and YFP-RGA (*B*), using the firefly *LUC* gene under the control of the wild-type version of the TCS element, and the constitutively expressed *Renilla* luciferase (*REN*) for normalization. The values represent the ratio between both luciferase activities and are the average of three biological replicates. Error bars are the standard deviation. The lower panel contains the western-blot analysis of protein samples corresponding to equal mixtures from the three leaves used for the LUC assays. Asterisks mark unspecific bands; arrowheads mark YFP-RGA (upper) and YFP-GAI (lower). Red colored panels are blots stained with Ponceau solution and serve as loading controls.
(TIF)

S1 Table ChIP-seq peaks of RGA-GFP. Only those peaks that can be assigned to a particular AGI code have been listed.
(XLSX)

S2 Table Motif occurrence analysis in RGA ChIP peaks. Analysis performed with motifs from "MotifCollection1" and sites from "BindingSites_MotifScanner1" on 310 sequences. Expected motif frequencies from "MotifNumericMap_expected". Statistical significance was evaluated using a binomial test with p-value threshold = 0.05 (Bonferroni-corrected threshold = 1.9E-4 considering all 254 motifs tested)
(XLSX)

S3 Table Known DELLA interactors that belong to the TF families with enriched cis elements in the RGA ChIP peaks. (XLSX)

S4 Table Differentially expressed genes in response to CK or Dexamethasone (35S::ARR1-GFP-GR seedlings) in the presence of paclobutrazol or paclobutrazol+GA. (XLSX)

S5 Table Oligonucleotides used as primers in this study. (XLSX)
