# Supplementary figures and images for: Genome Wide Binding Site Analysis Reveals Transcriptional Coactivation of Cytokinin-Responsive Genes by DELLA Proteins
Source: PLoS Genet. 2015 Jul 2;11(7):e1005337. doi: 10.1371/journal.pgen.1005337 (PMC4489807; doi:10.1371/journal.pgen.1005337)

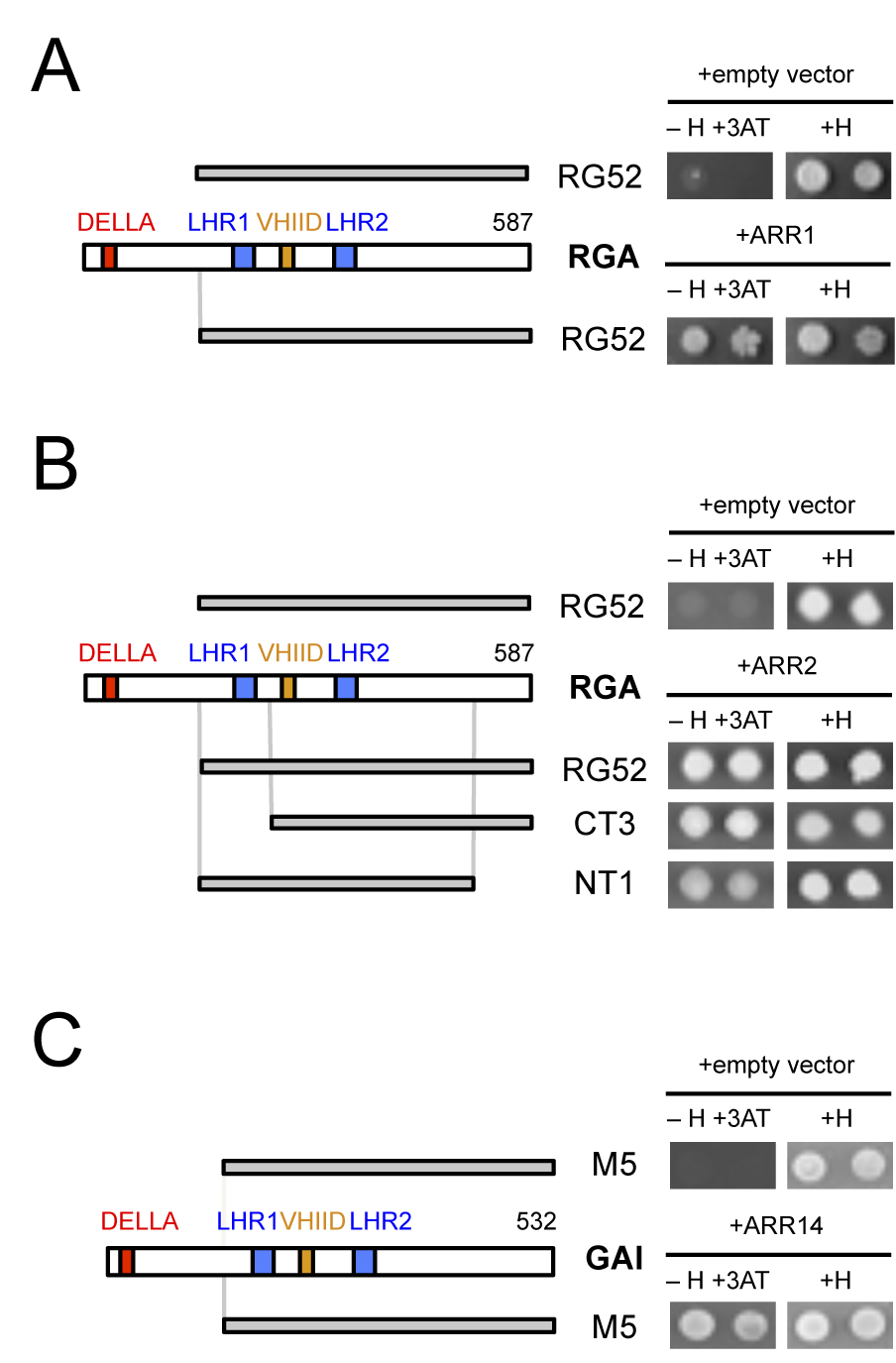

Supplement: S1 Fig — (A) Y2H assay of the interaction between ARR1 and a truncated version of RGA without the DELLA domain. (H, Histidine; 3-AT, 5mM 3-aminotriazol) (B) Y2H assay of the interaction between ARR2 and truncated versions of RGA. (H, Histidine; 3-AT, 5mM 3-aminotriazol) (C) Y2H assay of the interaction between ARR14 and a truncated version of GAI without the DELLA domain. (H, Histidine; 3-AT, 5mM 3-aminotriazol) (TIF) [file pgen.1005337.s001.tif]

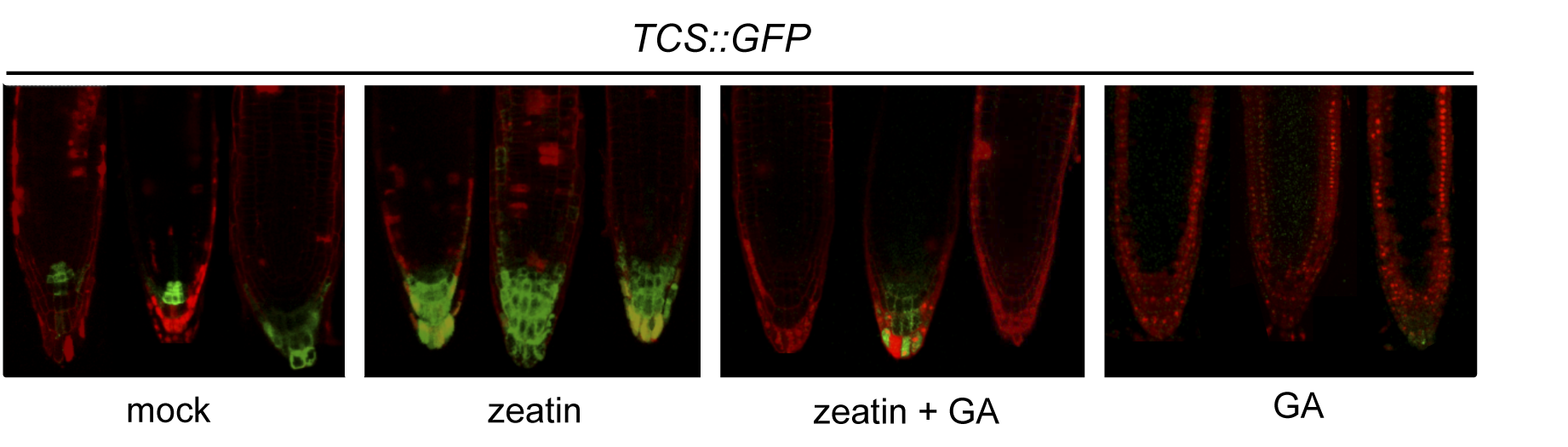

Supplement: S2 Fig — Expression in Arabidopsis roots of GFP under the control of the CK- and ARR1-responsive TCS element, after treatments with 0.5 μM trans-zeatin and 1 μM GA4. Several individuals are shown, to complement the information of Fig 3A. (TIF) [file pgen.1005337.s002.tif]

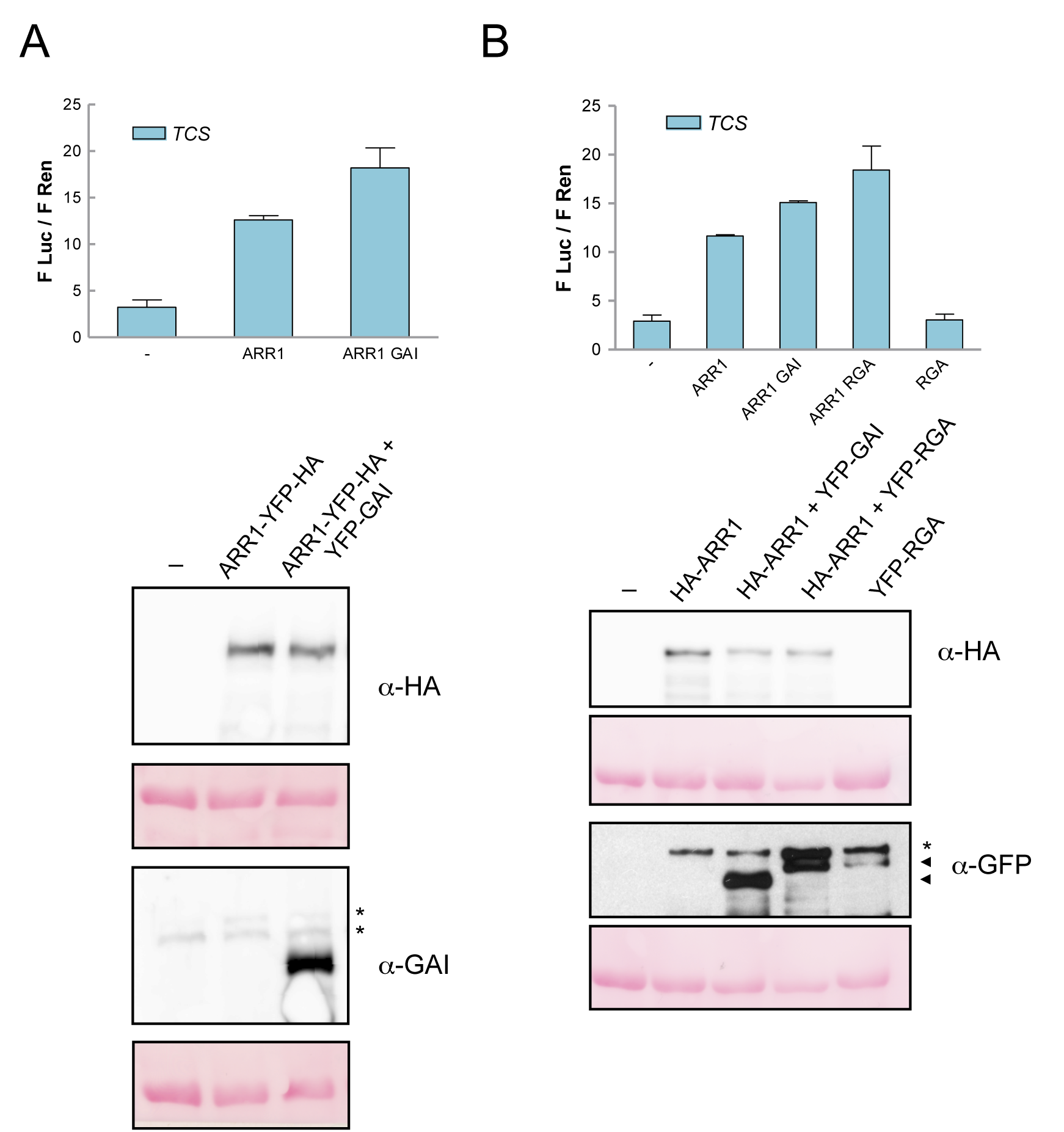

Supplement: S3 Fig — Luciferase assays in N. benthamiana leaves agroinfiltrated with ARR1-YFP-HA and YFP-GAI (A), and with HA-ARR1, YFP-GAI and YFP-RGA (B), using the firefly LUC gene under the control of the wild-type version of the TCS element, and the constitutively expressed Renilla luciferase (REN) for normalization. The values represent the ratio between both luciferase activities and are the average of three biological replicates. Error bars are the standard deviation. The lower panel contains the western-blot analysis of protein samples corresponding to equal mixtures from the three leaves used for the LUC assays. Asterisks mark unspecific bands; arrowheads mark YFP-RGA (upper) and YFP-GAI (lower). Red colored panels are blots stained with Ponceau solution and serve as loading controls. (TIF) [file pgen.1005337.s003.tif]
